# Augmented reality navigation with intraoperative 3D imaging vs fluoroscopy-assisted free-hand surgery for spine fixation surgery: a matched-control study comparing accuracy

**DOI:** 10.1038/s41598-020-57693-5

**Published:** 2020-01-20

**Authors:** Adrian Elmi-Terander, Gustav Burström, Rami Nachabé, Michael Fagerlund, Fredrik Ståhl, Anastasios Charalampidis, Erik Edström, Paul Gerdhem

**Affiliations:** 1 Department of Clinical Neuroscience, Karolinska Institutet, Stockholm, Sweden; 2 Department of Neurosurgery, Karolinska University Hospital, Stockholm, Sweden; 3 Department of Image Guided Therapy Systems, Philips Healthcare, Best, the Netherlands; 4 Department of Neuroradiology, Karolinska University Hospital, Stockholm, Sweden; 5 Department of Clinical Sciences, Intervention and Technology (CLINTEC), Karolinska Institutet, Stockholm, Sweden; Department of Orthopedics, Karolinska University Hospital, Stockholm, Sweden

**Keywords:** Three-dimensional imaging, Neuromuscular disease

## Abstract

This study aimed to compare screw placement accuracy and clinical aspects between Augmented Reality Surgical Navigation (ARSN) and free-hand (FH) technique. Twenty patients underwent spine surgery with screw placement using ARSN and were matched retrospectively to a cohort of 20 FH technique cases for comparison. All ARSN and FH cases were performed by the same surgeon. Matching was based on clinical diagnosis and similar proportions of screws placed in the thoracic and lumbosacral vertebrae in both groups. Accuracy of screw placement was assessed on postoperative scans according to the Gertzbein scale and grades 0 and 1 were considered accurate. Procedure time, blood loss and length of hospital stay, were collected as secondary endpoints. A total of 262 and 288 screws were assessed in the ARSN and FH groups, respectively. The share of clinically accurate screws was significantly higher in the ARSN vs FH group (93.9% vs 89.6%, p < 0.05). The proportion of screws placed without a cortical breach was twice as high in the ARSN group compared to the FH group (63.4% vs 30.6%, p < 0.0001). No statistical difference was observed for the secondary endpoints between both groups. This matched-control study demonstrated that ARSN provided higher screw placement accuracy compared to free-hand.

## Introduction

Compared to conventional free-hand (FH) surgical technique, computer-assisted navigation using intraoperative 3D imaging has been shown to improve screw placement accuracy and reduce complications due to screw misplacements^1^. Moreover, improved accuracy has also been shown in more challenging conditions, such as scoliosis surgery, where it may be of even greater importance^2^. Consequently, navigation also reduces the frequency of postoperative revision surgery compared to FH surgery^3^.

Although several studies have compared intraoperative image guidance to free-hand (FH) technique, the evidence in favor of navigation is still limited. In a recent systematic review, Chan *et al*., found only four studies comparing computed tomography (CT) guidance with free-hand methods head-to-head, including one small (10 patients in each group, 169 screws in total) randomized study^4^. Overall, the reviewers found only moderate level evidence showing that CT guidance has lower breach rates than FH, while screw-related complication rates were conflicting at 0% in CT navigation compared with 0%–1.7% in FH groups^5^. In a more recent retrospective study comparing O-arm navigation to FH, Wang *et al*. demonstrated higher pedicle screw accuracy and lower total OR-time for the navigation group^6^. However, the introduction of new navigation technologies requires additional data reflecting the value of navigation compared to FH technique both regarding accuracy and secondary clinical outcome measures.

Most commercially available surgical navigation systems are based on infrared cameras detecting reflective spheres attached to the spine of the patient via a dynamic reference frame. A video-based system, providing augmented reality surgical navigation (ARSN) with intraoperative 3-dimensional (3D) imaging is a novel form of computer-assisted navigation^7^. It has been shown to be accurate, safe and effective for pedicle screw placement^8^. However, ARSN has not yet been directly compared to the conventional FH technique in a clinical setting.

Therefore, the objective of the current study was to evaluate pedicle screw placement accuracy as well as other clinical aspects of ARSN, compared to FH technique.

## Materials and Methods

### Patient population

This HIPAA-compliant study was approved by the Karolinska Institutet medical ethical committee and was conducted according to the relevant guidelines regulations. Informed consent was obtained from the patients in the ARSN group and waived for the retrospective control group by the research ethics board which approved this study. Representing the intervention group were 20 patients who had previously undergone spine surgery with pedicle screw placement using ARSN as part of a prospective clinical study. The control group of 20 patients, was retrospectively selected from recent FH cases performed by the same spine surgeon that performed the navigated surgery. Care was taken to balance the proportion of thoracic to lumbosacral pedicle screws in the material. Thoracic pedicles are narrower than lumbar, and since accuracy measured using the Gertzbein scale correlates with the width of the pedicles, the proportion of screws placed in the thoracic spine will influence the overall outcome^9^. The groups were matched based on the clinical diagnosis, and the proportion of screws placed in the thoracic versus lumbosacral spine. In the ARSN group, there were 13 scoliosis, 2 kyphosis and 5 other pathologies. Similarly, in the FH group there were 13 scoliosis, 2 kyphosis, and 5 other conditions.

### Surgical workflow

All 40 procedures were performed by the same orthopedic spine surgeon, with assistance from senior spine surgeons and trained staff. The orthopedic spine surgeon had more than 18 years of experience in spinal surgery with FH technique, but no former experience with navigation systems.

During all procedures, the patients were under general and local anesthesia and placed in the prone position. A midline approach was used to expose the spine followed by muscle detachment along the lamina and spinous processes.

**Figure 1:**
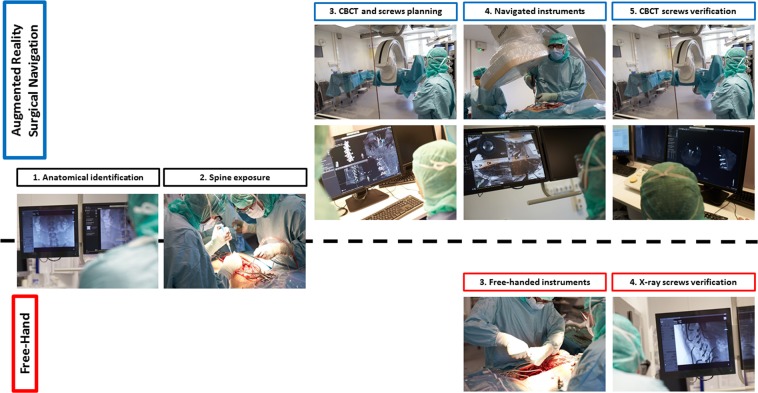
Surgical workflow comparison between augmented reality surgical navigation (ARSN) and free-hand (FH) techniques for spine fixation. Anatomical identification and exposure is similar for both groups (steps 1 and 2). ARSN require an intraoperative cone beam CT (CBCT) is remotely acquired behind a lead shield for screw planning and sizing to ensure an optimized placement (step 3 of ARSN). Subsequently, instruments are navigated (a drill in the example of step 4) and a CBCT for screw placement verification is performed (step 5 ARSN). In the FH group, instruments are manually used relying on visual and tactile feedbacks (step 3 FH). X-rays are performed for screw verification (step 4 FH).

**Figure 2:**
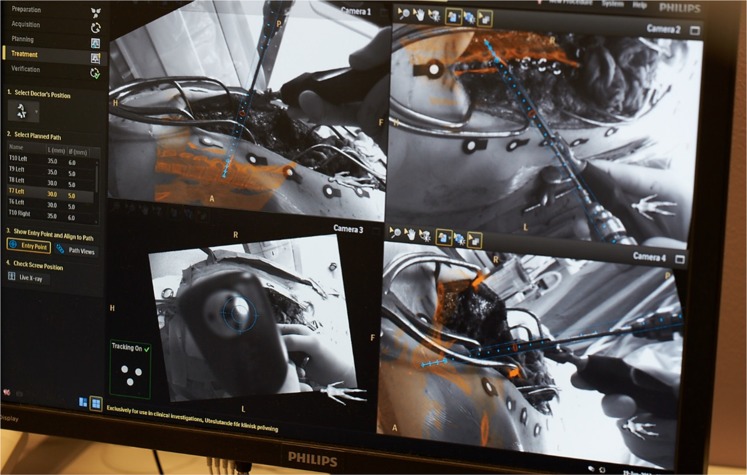
The surgical interface during navigated surgery is depicted. Each quadrant shows the surgical area at an angle, with the suggested instrument path in blue and underlying bone anatomy superimposed on the skin.

The ARSN system described in this article is currently a research prototype not available for commercial use. The surgeries in the ARSN group, were performed in a hybrid operating room (Allura Flexmove, Philips, Best, The Netherlands) with a ceiling-mounted robotic C-arm with integrated video cameras within the x-ray detector frame for AR navigation^8^. After exposure of the spine, adhesive skin markers were placed for patient tracking and a 3D cone beam CT (CBCT) was performed to image the region that required spinal instrumentation. Planning of pedicle screw trajectories was performed based on the CBCT images and automatic spine segmentation^10^. The screws were one by one activated in the software. The C-arm rotated to the proper position for each screw to display the path to follow during navigation. The bone entry-point was identified with augmented reality and an awl was used to create an initial hole. A gearshift or power drill was then used to navigate along the planned path and create a pilot hole, before navigated screw placement using the ARSN system. One camera of the ARSN system provided a “bulls-eye” view along the screw axis while the other 3 cameras provided guidance for alignment of the instruments during navigation^11^. After placement of the screws an intraoperative CBCT was performed to assess screw placement before wound closure^11^. In the FH group, the screws were placed based on anatomical landmarks and x-ray fluoroscopy was used when necessary. Figure 1 depicts the difference in surgical workflow between both groups, Fig. 2 highlights the interface during navigated surgery.

### Radiological and clinical evaluation

**Figure 3:**
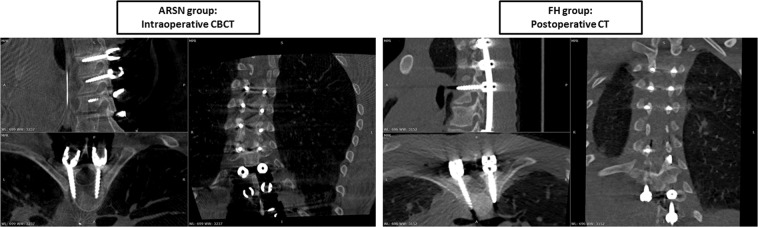
Examples of multi-planar images displayed according to each screw axis for screw position assessment. On the left, rating performed on an intraoperative cone beam CT of a patient treated with augmented reality surgical navigation (ARSN). On the right, rating performed on a postoperative CT of a patient treated with the free-hand (FH) technique.

The pedicle and iliac screw placements were assessed for cortical breaches on the intraoperative 3D CBCT and postoperative CT images for the ARSN and the FH groups, respectively. Three independent reviewers, two neuroradiologists and one orthopedic spine surgeon, performed the assessment. The Gertzbein scale was used to evaluate the clinical accuracy: grade 0 (no cortical breach), grade 1 (0–2 mm breach, minor perforation including cortical encroachment), grade 2 (>2–4 mm breach, moderate breach) and grade 3 (>4 mm breach, i.e. severe displacement)^12^. Screws assessed as Gertzbein grade 0 and 1 were considered accurately placed. Direction of breaches larger than 2 mm were documented. The assessment was performed in multi-planar views along the axis of the screw. Figure 3 depicts an example of a screw assessed under CBCT and CT for the ARSN and free-hand group, respectively^13^.

The procedural time, length of hospital stay and blood loss were collected for comparison between the groups.

### Statistical analysis

Descriptive summary statistics are expressed as mean (±standard deviation), or frequency (percentage). One-sided and two-sided Fisher’s exact test and Welch’s t-test were used where applicable for categorical and continuous data comparison, respectively. Statistical significance was set at p < 0.05. Statistical analysis was performed using the statistics toolbox of Matlab (Mathworks, Natick, MA).

## Results

A summary of patient demographics and clinical diagnosis is detailed in Table 1. Gender, age, weight, and height were statistically comparable in both groups except for body-mass index.

**Table 1:** Patient demographics and surgical characteristics comparison between the augmented reality surgical navigation (ARSN) and free-hand (FH) groups.

Characteristics	ARSN group	FH control group	p-value
Total amount of patients	20	20	
Male-Female	9–11 (45%–55%)	9–11 (45%–55%)	1
Age (years)	**30.0 ± 19.4**	**36.6 ± 23.2**	**0.3765**
Weight (kg)	**58.8 ± 7.0**	**66.9 ± 17.5**	**0.0666**
Height (cm)	**172 ± 8**	**170 ± 13**	**0.4655**
BMI (kg/m^2^)	**19.8 ± 2.1**	**23.2 ± 5.5**	**<0.05**
**Primary diagnosis**			**1**
Scoliosis	13 (65%)	13 (65%)	
Kyphosis	2 (10%)	2 (10%)	
Other	5 (25%)	5 (25%)	

BMI: body-mass index.

**Figure 4:**
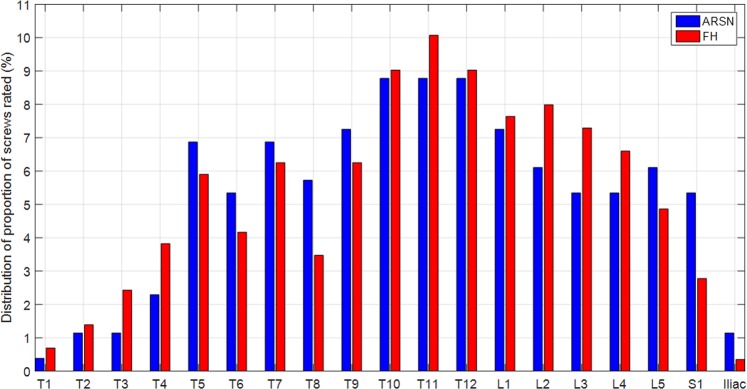
Distribution of screws rated for placement accuracy in the augmented reality surgical navigation (ARSN) and free-hand groups per anatomical region. There was no statistical difference in the proportion of screws placed in the thoracic spine or at each level between both groups.

A total of 262 and 288 screws were radiologically assessed in the ARSN and FH groups, respectively. The proportion of thoracic pedicle screws was comparable between the groups (ARSN group: 63.4% vs FH group: 62.5%, p = 0.86). No difference was observed per anatomical region. Figure 4 depicts the distribution of screws that were assessed for placement accuracy per anatomical region.

Table 2 details the amount and proportion of screws and the respective clinical accuracies for both the ARSN and FH groups. The clinical accuracy of the ARSN group was significantly higher compared to the FH group (ARSN group: 93.9% vs FH group: 89.6%, p < 0.05). The proportion of grade 0 screws was twice as high in the ARSN group compared to the FH group (63.4% vs 30.6%, p < 0.0001). No screws were considered severely misplaced (i.e. grade 3) in either group. A minority of grade 2 breaches were medial, with no statistical difference between groups (AR group: 2/16 i.e. 12.5% vs FH group: 5/30 i.e. 16.7%, p = 0.535).

**Table 2:** Radiological assessment of placed screws for the augmented reality surgical navigation (ARSN) and free-hand (FH) groups.

Screw assessment	ARSN group	FH control group	p-value
Total number of screws rated	262 (100%)	288 (100%)	0.86
Thoracic spine	166 (63.4%)	180 (62.5%)	
Lumbosacral spine and iliac	96 (35.6%)	108 (37.5%)	
**Screw placement grade**			
Grade 0	166 (63.4%)	88 (30.6%)	<0.0001
Grade 1	80 (30.5%)	170 (59.0%)	<0.0001
Grade 2	16 (6.1%)	30 (10.4%)	05
Grade 3	0 (0.0%)	0 (0.0%)	1
Accuracy	246 (93.9%)	258 (89.6%)	<0.05

**Figure 5:**
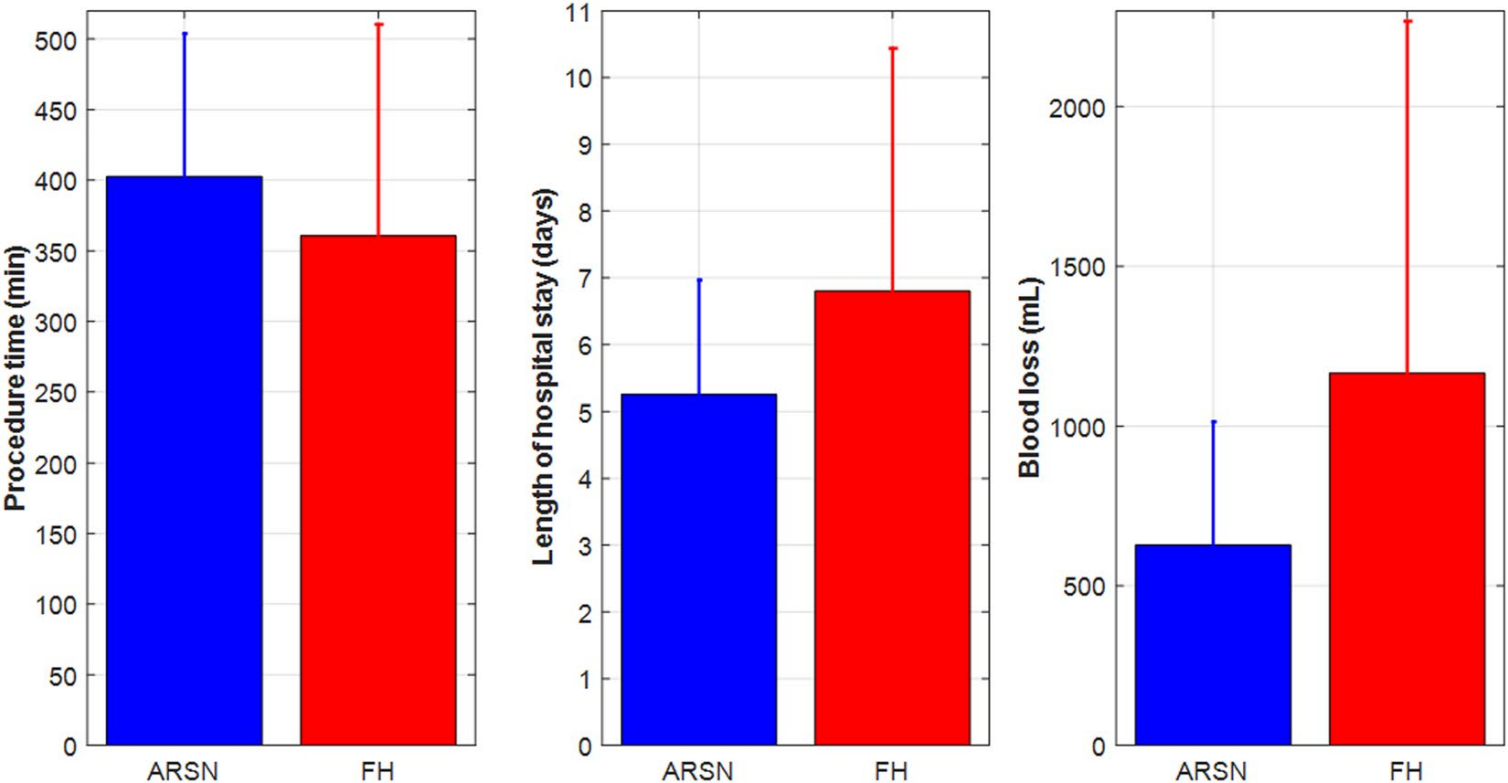
Comparison of mean ± standard deviation of procedure time (subfigure on the left), length of hospital stay (subfigure in the middle), and blood loss (subfigure on the right) between the augmented reality surgical navigation (ARSN) and the free-hand groups. None of these secondary endpoints indicated statistical difference.

There were no statistical differences between the groups in procedural time (ARSN group: 403 ± 101 min vs FH group: 361 ± 150 min, p = 0.3078), length of hospital stay (ARSN group: 5.3 ± 1.7 days vs FH group: 6.8 ± 3.6 days, p = 0.096) or in blood loss (ARSN group: 628 ± 386 mL vs FH group: 1165 ± 1103 mL, p = 0.0569), as depicted in Fig. 5.

## Discussion

This is the first study comparing the clinical accuracy for spinal pedicle screw placement, between augmented reality surgical navigation (ARSN) and free-hand (FH) techniques. The study demonstrates that ARSN has a higher accuracy than FH for pedicle screw placement (93.9% vs 89.6%). This is in line with recent meta-analyses comparing other types of navigation systems to conventional surgical methods, showing increased accuracy when using navigation^1,5^.

Navigation has been demonstrated to have the largest impact on accuracy in complex deformity surgeries, especially in the neuromuscular type of scoliosis^14^, and in the thoracic spine where the pedicles can be very small^15^. Narrow thoracic pedicles, altered by scoliosis, can provide a formidable challenge in spinal instrumentation^8,9,16^. Studies showing smaller differences in accuracy are mainly studies without matching per diagnosis, or proportion of screws placed in the thoracic and lumbar spine. For example, the data by Noschenko *et al*. had twice as many deformity cases in the navigation group compared to the FH group^17^. This potentially explains the small difference in accuracy between both groups as deformity cases are more challenging. The studies by Laudato *et al*.^18^ and Shin *et al*.^19^. showed non-significant differences in accuracy, but had a larger proportion of screws in the thoracic spine in the navigation group compared to the FH group.

Despite using ARSN, 16 screws (6.1%) were graded as Gertzbein grade 2. We argue that this reflects a shortcoming of the Gertzbein grading system in a clinical context, since pedicle screws are purposefully chosen to be wider than the pedicle when possible^20^. In addition, the ARSN system enabled us to place pedicle screws in very narrow pedicles, which would otherwise have been skipped or fixed with hooks. Placing a screw larger than the pedicle width, will inevitably result in a Gertzbein grade 2 even if the screw placement is accurate. This phenomenon is reflected in the significant decline of pedicle width-to-screw diameter ratio (average 1.3 ± 0.7) between Gertzbein grades 0–2. The pedicle to screw diameter ratios for Gertzbein grades 0, 1 and 2 were 1.4 ± 0.8, 1.0 ± 0.3, and 0.8 ± 0.2 respectively^8^. Only 3 screws (1.1%) in our material were intraoperatively revised due to unsatisfactory clinical placement.

A major difference between ARSN and infrared camera-based navigation systems is the different patient tracking methods. ARSN uses skin markers around the surgical area while infrared camera-based navigation typically uses markers on a reference frame clamped to a vertebra. For optimal accuracy, it is suggested that the reference frame should be attached to the navigated vertebra, and it has been shown that accuracy decreases with distance from the reference frame. In fact, Jin *et al*. demonstrated that a distance of 2 vertebral levels, between the reference frame and the instrumented level, doubles the risk of screw misplacement and a distance of 3 vertebral levels or more, quadruples it^1^. Even though Urbanski *et al*.^21^ had their intervention and control groups matched for diagnosis as well as for the proportion of screws placed in the thoracic spine (73.8% vs 77.6%), they did not reach significant difference in accuracy. They argued that the lack of difference in accuracy between the groups was due to the reference frame being mounted at T8-T9, up to 15 cm from the upper thoracic levels of T1-T2. Consequently, the misplacement rate in the upper and mid thoracic levels T1-T5 was 17% in the navigation group, almost twice as much in comparison to the FH group with a misplacement rate of 8%. The optical tracking system used by the ARSN, identifies not only the individual adhesive skin markers, but also creates a 3D point pattern based on their relative positions to each other^22^. This is used as a virtual reference grid for patient tracking and is designed to have a redundancy whereby it accepts occlusion or removal of several adhesive skin markers, if a minimum of five are still in place. This feature allows maintained navigation accuracy despite manipulation during surgery. Since the virtual reference grid used in the ARSN system does not have a specific “index vertebra”, the accuracy in the surgical field is uniform.

The spine surgeon in this study, had more than 18 years of experience with FH technique and no prior experience with any kind of navigation system before the start of the ARSN study. Therefore, it can be expected that the results are influenced by a learning curve for using the navigation system and that the difference in accuracy between the groups could increase with increased experience. In fact, it was demonstrated by Rivkin *et al*. that the accuracy continuously increases for every 30 cases from 86.8% to 98.9% after 270 cases^23^. They concluded that at least 30 cases are needed to reach an acceptable accuracy of around 95%.

Although intraoperative 3D imaging can increase the accuracy of pedicle screw placement, this technique may increase the total radiation exposure to staff and patients compared to fluoroscopy. Nonetheless, we have previously shown that the occupational exposure can be minimized using ARSN, as the navigation part of the surgery is radiation free and the staff can use protective shielding during CBCT acquisition. The average staff exposure in the ARSN group was 0.21 ± 0.06 μSv^24^. However, intraoperative 3D imaging can potentially increase the patient’s radiation exposure. Even though a CBCT acquisition increases the patient radiation dose compared to fluoroscopy, the dose is on average lower than that of a single spine CT^25^. Furthermore, intraoperative 3D imaging may prevent repeat surgeries and reduce the need for follow-up imaging, thereby reducing the cumulative patient dose. Using a low-dose CBCT-protocol could also reduce the patient radiation exposure^24^.

Although not evaluated in this study, the use of intraoperative 3D imaging itself has the potential to reduce screw malposition rate. When procedures are performed using FH technique, a mobile C-arm is used to perform 2D fluoroscopic images to check screw placement intraoperatively. However, this type of imaging cannot create axial views and therefore mediolateral breaches can only be identified through an anteroposterior projection view. When comparing 2D radiographic imaging vs 3D CT imaging, the sensitivity of a breach detection in non-deformity cases is 74%^26^ and decreases to 52% in deformity cases^27^.

There was no statistically significant difference in procedure time in the ARSN group compared to the FH group but there was a trend towards longer times in the former. We believe that any difference in surgical time will shrink with increased experience with the ARSN technology. It could be argued that ARSN requires additional time for system set-up, attaching the skin markers, planning, and registration resulting in prolonged OR-time. However, we have demonstrated that the time required for CBCT-acquisition, screw path planning and verification amounted to 8% (median of 8 min) of the procedure time^11^. Unfortunately, the retrospectively collected data of the control group only included the total procedure time and the fluoroscopy time could not be calculated for comparison. Studies on procedural time using navigation have shown conflicting results, demonstrating both longer^14,15,28^ and shorter times^6,29^.

There was a trend towards shorter length of hospital stay and lower blood loss in the navigation group in this study; although not statistically significant. Studies which have compared length of hospital stay and blood loss have shown as well non-significant trends towards a decrease compared to free-hand^28–30^. Our sample size is too small to detect any such differences in these secondary endpoints.

## Limitations

The ARSN system is a hybrid-OR based system. It could be argued that access to hybrid ORs may be limited, or non-existent, in some centers and that the system can only be used for one patient at the time compared to a mobile imaging device. Furthermore, one obese patient (body mass index of 37) in the prospective study could not be treated by ARSN since proper isocentering of the spine could not be achieved resulting in cropped 3D visualization and there was limited space between the detector and the patient for navigation. However, this limitation extends to other imaging-based systems as well^31^.

Limitations of this study include the small sample size and the fact that it is not a randomized-control trial but a comparison of a prospective group vs a retrospectively matched-control group. Some variables could not be retrospectively collected for comparison. A randomized study of specific subtypes of spinal pathology and with a larger sample size would be required to achieve higher clinical evidence.

## Conclusion

Augmented reality surgical navigation with intraoperative 3D imaging in a hybrid operating room demonstrated a statistically higher screw placement accuracy compared to the free-hand technique in a cohort of mostly spinal deformity cases. Procedure time, length of hospital stay, and blood loss did not show any statistical difference between surgical techniques.
